# Editorial: Advances in hybrid and application-driven BCI systems

**DOI:** 10.3389/fnhum.2024.1498196

**Published:** 2024-10-22

**Authors:** Minkyu Ahn, Bradley Jay Edelman, Bin He, Gernot R. Müller-Putz, Florian Röhrbein

**Affiliations:** ^1^School of Computer Science and Electrical Engineering, Handong Global University, Pohang, Republic of Korea; ^2^Max Planck Institute of Psychiatry, Munich, Germany; ^3^Max Planck Institute for Biological Intelligence, Planegg, Germany; ^4^Carnegie Mellon University, Pittsburgh, PA, United States; ^5^Institute of Neural Engineering, Graz University of Technology, Graz, Austria; ^6^Department of Computer Science, Chemnitz University of Technology, Chemnitz, Germany

**Keywords:** reproducibility, replicability, brain-computer interfaces, human neuroscience, brain-machine interface

This Research Topic features one mini-review and three original research articles that focus on advancements in EEG-based Brain-Computer Interface (BCI) technology. A mini review provides an overview on BCI spelling systems, discussing their underlying paradigms and highlights hybrid spellers. The research articles explore a range of applications, including AttentionCARE, a BCI designed to aid adolescents at high risk for depression; a hybrid BCI system combining eye-tracking and steady-state visual evoked potentials (SSVEP) for controlling a robotic arm in virtual reality; and a method for classifying EEG signals using fewer channels to enhance the performance of motor imagery BCI systems:

The review of Maslova et al. presents the most popular paradigms used as BCI speller systems. There are three main types of BCI-spellers: P300, motor imagery (MI), and steady-state visual evoked potential (SSVEP). While each type has its own limitations, hybrid BCI-spellers combine the strengths of multiple types. They can improve accuracy and reduce the training period required for users to become proficient.

The study by Gall et al. looks at a brain-computer interface called AttentionCARE, which helps adolescents at high risk for depression by using augmented reality and EEG to change how they focus on negative emotions. It mainly targets adolescents with depressed mothers. The results show that AttentionCARE can reliably give feedback on attention to negative emotions, which is important for future treatments. This research helps ensure that such brain-computer interfaces are reliable and can be used in future interventions.

Guo et al. reported a study of hybrid BCI using SSVEP and eye-tracking in virtual reality environment for virtual robotic arm control. Robotic arm control has been previously reported using motor imagination for reaching and grasping tasks and continuous movement and tracking from sensorimotor rhythm, and using motor execution. SSVEP has been also previously used for robotic arm control in a BCI setup. In this study, the authors used a hybrid BCI integrating SSVEP and eye-tracking to further improve the performance. They also tested the system in a virtual reality environment. Results show that with integration of SSVEP and eye-tracking, the number of commends was increased from 4 to 8, as compared to previous work on physical robotic arm control using motor imagination. While further testing in a physical environment is desirable, this work suggests the merits of hybrid BCI through integrating SSVEP and eye-tracking.

Long et al. address the challenge of classifying EEG signals in BCI systems due to high dimensionality from excessive channels. They proposed the Combination of the Euclidean-space Alignment (EA) and Optimized Subband Regularized Common Spatial Pattern to enhance motor imagery BCI systems' performance with fewer channels. Using Dataset1 from BCI Competition IV (2008) with nine subjects and 22 channels, and Dataset2, self-collected from 10 healthy subjects with eight channels, they demonstrated reasonable classification accuracies (the 78.01% accuracy and a kappa coefficient of 0.54 for Dataset1 and 59.77% accuracy and a kappa coefficient of 0.34 for Dataset2), outperforming recent methods despite fewer channels. Therefore, the proposed method is expected to reduce the dependence on the number of channels and samples, simplifying BCI models and enhancing system user-friendliness.

In summary, these studies offer valuable insights into the progression of BCI technology, reviewing BCIs as communication devices, replicability in clinical settings, and improving control systems for robotic devices. The research highlights advancements in hybrid systems and innovative classification methods that enhance both the accuracy and usability of BCI systems, paving the way for more practical and reliable applications in both clinical and real-world environments.

